# Association between COVID-19 infection, elevated C-reactive protein, and neuropsychiatric symptoms in individuals with metabolic and cardiovascular comorbidities

**DOI:** 10.1007/s11011-026-01936-3

**Published:** 2026-07-20

**Authors:** Gabriel S. Mondo, Lucas C. Pedro, Camila O. Arent, Larissa C. Pereira, Jéssica L. Fernandes, Amanda L. Maciel, Luciane B. Ceretta, Zuleide Maria Ignácio, Gislaine Zilli Réus

**Affiliations:** 1 Translational Psychiatry Laboratory, Graduate Program in Health Sciences, Universidade do Extremo Sul Catarinense (UNESC), Criciúma, SC Brazil; 2 Graduate Program in Public Health, Universidade do Extremo Sul Catarinense (UNESC), Criciúma, SC Brazil; 3 Laboratory of Physiology, Pharmacology, and Psychopathology, Graduate Program in Biomedical Sciences, Federal University of the Southern Frontier, Chapecó, SC Brazil; 4 Translational Psychiatry Laboratory Graduate Program in Health Sciences, Universidade do Extremo Sul Catarinense, ZipCode, Criciúma, 1105, 88806-000 SC Brazil

**Keywords:** Inflammation, COVID-19, Anxiety, Systemic arterial hypertension, Diabetes, Major depressive disorder

## Abstract

Elevated serum inflammatory markers are associated with neuropsychiatric symptoms. This process is potentially more intense in individuals with chronic comorbidities such as obesity, systemic arterial hypertension (SAH), and diabetes mellitus (DM). Such conditions already establish a basal inflammatory state, which can be aggravated by acute viral infections, such as COVID-19, worsening neuropsychiatric outcomes. Given this panorama, this cross-sectional study aimed to analyze the association between the presence of pre-existing comorbidities, elevated plasma C-reactive protein (CRP) levels, and symptoms of stress, anxiety, and depression in individuals previously infected by SARS-CoV-2. The research included 350 participants: 114 in the post-COVID-19 group and 236 in the control group. The post-COVID-19 group had a higher prevalence of comorbidities, notably obesity (17.9%; *p* = 0.044), DM (13.3%; *p* < 0.01), and SAH (22.1%; *p* = 0.024). Furthermore, serum CRP levels were significantly higher in the post-COVID-19 group (*p* = 0.014) and correlated significantly with all comorbidities. The post-COVID-19 group presented higher stress (*p* = 0.02) and severity of depressive symptoms (*p* = 0.034). Specifically, the presence of SAH and obesity was associated with a significant increase in stress levels, depression, and anxiety severity (*p* = 0.027). In conclusion, the results demonstrate that individuals with pre-existing comorbidities, besides being more prevalent in the post-COVID-19 group, exhibit a higher systemic inflammatory state (high CRP), associated with the exacerbation of neuropsychiatric symptoms.

**Graphical Abstract:**

**Figure:**
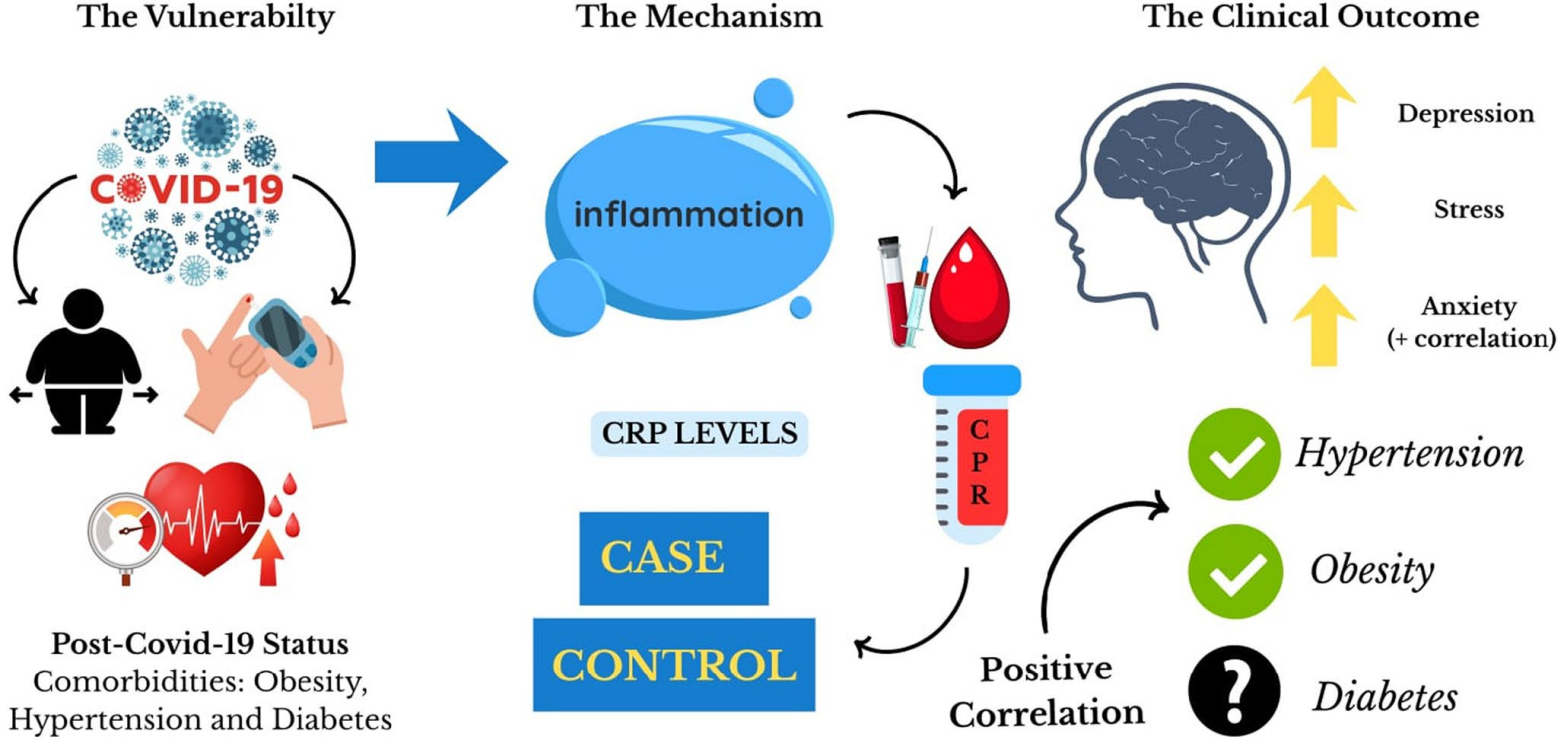
Graphical Abstract of the interaction between SARS-CoV-2 infection, systemic inflammation, and neuropsychiatric outcomes. The diagram illustrates that individuals in the post-COVID-19 group, particularly those with obesity and systemic arterial hypertension (SAH), exhibit significantly elevated C-reactive protein (CRP) levels. This persistent inflammatory state correlates positively with the increased severity of depressive symptoms, anxiety, and stress. The findings highlight a synergistic effect between viral sequelae and cardiometabolic comorbidities in driving mental health repercussions.

## Introduction

The COVID-19 pandemic, caused by the SARS-CoV-2 virus, emerged as one of the greatest public health challenges of the 21st century, with impacts that extend beyond acute respiratory manifestations (World Health Organization (WHO) 2020; Pedro et al. 2025). Beyond direct morbidity and mortality, the infection has been associated with long-term systemic and neuropsychiatric sequelae (Rogers et al. 2020). Furthermore, individuals with pre-existing metabolic and cardiovascular comorbidities, such as diabetes mellitus (DM), systemic arterial hypertension (SAH), and obesity, constitute a group of higher vulnerability, presenting an increased risk for severe forms of COVID-19 and worse outcomes (Armstrong et al. 2022).

The underlying pathophysiology of this vulnerability involves a chronic, low-grade inflammatory state, common to these comorbidities, which can be drastically exacerbated by the immune response to viral infection (Hussman 2020). To quantify this inflammatory process, C-reactive protein (CRP), an acute-phase inflammatory marker produced by the liver in response to interleukin-6 (IL-6), has been widely used (Volanakis 2001). Notably, elevated CRP levels are associated not only with the severity of COVID-19 (Luo et al. 2020; Miyata et al. 2022) but also with the pathophysiology of neuropsychiatric disorders, such as depression and anxiety (Howren et al. 2009; Osimo et al. 2019).

Accordingly, recent studies show a high prevalence of depressive, anxious, and stress symptoms in COVID-19 survivors, often correlated with elevated inflammatory markers (Mazza et al. 2020; de Azevedo Cardoso et al. 2023). However, the specific interaction among prior SARS-CoV-2 infection, a persistent inflammatory profile (measured by CRP), and mental health in individuals with a pro-inflammatory background due to metabolic and cardiovascular comorbidities remains underexplored. In this context, CRP serves as a robust, standardized, and clinically accessible surrogate marker of the upstream inflammatory pathway, allowing investigation of its specific correlation with affective and cognitive symptoms (Palmer et al. 2024). Consequently, understanding this relationship is crucial for the development of integrated rehabilitation strategies that address both the physical and mental sequelae post-COVID-19. Therefore, this study aims to investigate the statistical associations between a history of COVID-19 infection, circulating CRP levels, and the prevalence and severity of neuropsychiatric symptoms in individuals with pre-existing metabolic and cardiovascular comorbidities. By exploring these interrelationships, we seek to clarify how systemic inflammatory markers, such as CRP, correlate with mental health outcomes in this vulnerable clinical population.

## Materials and methods

### Study design

This is a cross-sectional study. This study is part of a larger investigation with the primary objective of assessing the impact of COVID-19 on mental health. In accordance with the Declaration of Helsinki, we conducted our clinical research ethically and responsibly. The study was undertaken when the objectives were important, and the risks were minimized, with potential benefits outweighing the risks. All participants were volunteers, with informed consent obtained in a clear and documented manner. We respected participants’ privacy and confidentiality and ensured that the research was approved by an independent ethics committee.

Further details have been published previously (de Azevedo Cardoso et al. 2023; Réus et al. 2024; Pedro et al. 2025; Borba et al. 2025; Barichello et al. 2025; Broseghini et al. 2025).

### Setting

The study took place between September 2020 and July 2021 in two locations: (1) Universidade do Extremo Sul Catarinense (UNESC, Criciúma, Brazil) and (2) Universidade Federal da Fronteira Sul (UFFS, Chapecó, Brazil). The Research Ethics and Human Research Committee approved the study at both institutions, under protocols 4,172,382 and 4,298,662, respectively. The study was only initiated after the participants signed the Free and Informed Consent Form (FICF).

### Participants

A convenience sampling strategy was adopted. The 350 individuals who participated were divided into two groups: (1) Case (*n* = 114), with a confirmed diagnosis of COVID-19 four to six weeks prior; and (2) Control (*n* = 236), with a negative rapid test for SARS-CoV-2. The inclusion criteria for both were: age 18 or older and residence in the South region of Brazil. Exclusion criteria included a previous diagnosis of bipolar disorder and physical/cognitive conditions that prevented participation. Cases were previously identified by the municipal health departments of Chapecó and Criciúma (Santa Catarina, Brazil) and referred to the study; the research team then contacted these individuals to assess eligibility. Additionally, case recruitment was supplemented through social media advertisements.

For the COVID-19 group, diagnosis was confirmed four to six weeks before study inclusion, a period adopted because the risk of viral transmission was lower. Controls were recruited from the immediate neighborhood of the included cases. This strategy was adopted to ensure that controls were as similar as possible to the cases in sociodemographic characteristics and environmental exposures, thereby minimizing potential confounding factors.

### Demographic characteristics and psychiatric assessment

The participants completed a sociodemographic questionnaire that included questions about sex, age, smoking status, and years of education. Additionally, we assessed medication use to treat or prevent COVID-19, DM, obesity, and systemic arterial hypertension via self-report. Individuals with COVID-19 infection were asked to rate the severity of their COVID-19 symptoms. Based on their responses, they were classified into two groups: (1) asymptomatic or mild symptom group: This group includes individuals who were diagnosed by a health professional and managed their symptoms at home; (2) group with moderate or severe symptoms: This group consists of individuals who required hospitalization, either in a general ward for moderate symptoms or in an intensive care unit for severe symptoms (de Azevedo Cardoso et al. 2023; Réus et al. 2024; Pedro et al. 2025; Borba et al. 2025; Barichello et al. 2025; Broseghini et al. 2025).

The severity of depressive symptoms was assessed using the Hamilton Depression Rating Scale (HAM-D). The instrument comprises 17 questions and is quantitatively classified by symptom severity. Scores above 25 points are considered characteristic of severely depressed individuals; scores between 18 and 24 points, moderately depressed individuals; and scores between 7 and 17 points, mildly depressed individuals. The total score is a discrete variable, with higher scores indicating greater severity (Hamilton, 1967).

The severity of anxiety symptoms was assessed using the Hamilton Anxiety Rating Scale (HAM-A), which consists of 14 items. Each item is evaluated on a scale from 0 to 4 for intensity (0 = absent; 2 = mild; 3 = moderate; 4 = severe). The sum of the scores for each item yields a total score ranging from 0 to 56. Its development was based on the principle that the more severe the manifestation of a pathology, the greater the number of characteristic symptoms that are present. If the number of symptoms is relatively high, counting the symptoms becomes a useful, reliable, and valid quantification tool (Hamilton 1959).

Stress levels were assessed using the Stress Symptom Inventory. The inventory consists of an adaptation of items from the Checklist-90-R Symptom Inventory (SCL-90-R) (Derogatis 1975). The SCL-90-R comprises 90 items, of which 24 were selected, with item scores ranging from 0 to 5 (Bertollo et al. 2020). Items expressing stress symptoms were selected and compared with stress items from the SCL-90-R (Lipp, 1994). The items identified were related to anxiety, depression, hostility, and somatization symptoms, but were not exclusively attributed to a psychiatric disorder.

Trained and qualified health professionals conducted the interview and blood collection. The interview was always conducted using data close to the date of blood collection, with no more than 15 days between the two. It is important to note that psychometric instruments were culturally adapted and psychometrically validated for the study population (de Azevedo Cardoso et al. 2023; Bertollo et al. 2020).

### C reactive protein analysis

For the biological analysis, 6 mL of blood was collected to obtain serum (for rapid antibody testing), and 4 mL was collected with EDTA anticoagulant for plasma. The blood was immediately centrifuged at 3,000 rpm for 10 min, and 10 µL of serum was used directly for the SARS-CoV-2 rapid antibody test (colloidal gold immunochromatography) following the manufacturer’s instructions (Leccurate, Lepu Technology). In addition, plasma samples were centrifuged and stored at -80 °C. CRP was subsequently measured in a supporting laboratory using the ultrasensitive immunoturbidimetric test.

### Statistical analysis

Statistical analysis was conducted using IBM SPSS Statistics 31.0. The normality of the variables was tested by the Shapiro-Wilk test. Continuous data with non-normal distributions were presented as medians (interquartile ranges) and compared using the Mann-Whitney U test. Categorical data (frequencies) were compared using Pearson’s chi-square test. The correlation between continuous variables was assessed using Spearman’s rank correlation coefficient (Mann 2003). Multivariate linear regression analyses were performed using CRP levels as the dependent variable to investigate independent associations with psychiatric symptoms. Separate models were constructed for depressive symptoms (HAM-D), anxiety symptoms (HAM-A), and stress levels. The analyses were adjusted for potential confounding variables, including sex, age, years of education, smoking status, COVID-19 medication use, and COVID-19 severity. Multivariate linear regression was performed only among individuals with a history of COVID-19. Standardized beta coefficients (β), confidence intervals, and p-values were reported. Statistical significance was considered at *p* < 0.05.

### Use of artificial intelligence tools

Artificial intelligence (AI) tools (Chat GPT 4.5 and Gemini 3.1 Pro) were used exclusively for English grammar verification, stylistic refinement, and text editing to enhance clarity and readability. The authors maintain full responsibility for the conceptualization, data analysis, interpretation of the literature, and the final content of the manuscript.

## Results

### Sociodemographic characteristics and comorbidity prevalence

Table 1 summarizes the sample characteristics. There were no statistically significant differences between the Case (post-COVID-19) and Control groups in sex (*p* = 0.765), age (*p* = 0.085), years of education (*p* = 0.349), and smoking status (*p* = 0.245), indicating that the groups were comparable on these variables. In individuals with COVID-19, the majority presented with mild disease or asymptomatic manifestations (79.8%) and reported using medication to treat COVID-19 and symptoms (83.3%) (Table 1). The prevalence of comorbidities was significantly higher in the Case group: obesity (17.9% vs. 10.2%; *p* = 0.044), diabetes mellitus (13.3% vs. 3.0%; *p* < 0.001), and systemic arterial hypertension (22.1% vs. 12.7%; *p* = 0.024).

**Table 1:** Sociodemographic and clinical characteristics between groups

Characteristics	Control (*n* = 236)	Case (*n* = 114)	*p*-value
Sex Female Male	157 (66.5%) 79 (33.5%)	74 (64.9%) 40 (35.1%)	0.765
Age	35 (47.00-25.25)	39 (52.50–28.00)	0.085
Years of education	16 (16.00–18.00)	15 (11.00–19.00)	0.349
Obesity Yes No	24 (10.2%) 212 (89.8%)	**20 (17.9%)** 92 (86.7%)	**0.044**
Diabetes Yes No	7 (3.0%) 229 (97.0%)	**15 (13.3%)** 98 (86.7%)	**< 0.001**
Hypertension Yes No	30 (12.7%) 206 (87.3%)	**25 (22.1%)** 88 (77.9%)	**0.024**
COVID-19 severity			-
Asymptomatic/mild symptoms		91 (79.8%)	
Moderate/severe symptoms		23 (20.2%)	
Use of medication for COVID-19			**< 0.001**
Yes	67 (29.1%)	95 (83.3%)	
No	163 (70.9%)	19 (16.7%)	
Smoking			0.245
Yes	21 (9.0%)	7 (6.1%)	
No	213 (91.0%)	107 (93.9%)	

Values are presented as n (%) for categorical variables or median (interquartile range) for continuous variables. *p*-values refer to comparisons between groups using the chi-square test (categorical variables) or the Mann–Whitney U test (continuous variables)

### COVID-19 severity and comorbidities

Results about COVID-19 severity and comorbidities, including hypertension, diabetes, and obesity, are represented in Table 2.

**Table 2:** Comorbidities in COVID-19 severity

Characteristics	Asymptomatic/mild symptoms *n* = 91 *n* (%) / Median (IQR)	Moderate/severe symptoms *n*=23 *n* (%) / Median (IQR)	χ^2^	Φ	OR	*p*-value
**Obesity**			0.025	0.02	1.10; 95% CI: 0.33–3.67	0.874
Yes	16 (17.6%)	4 (19.0%)				
No	75 (82.4%)	17 (81.0%)				
**Diabetes mellitus** **Type 2**			4.650	0.20	3.42, 95% CI: 1.12–10.46	**0.031**
Yes	9 (9.9%)	**6 (27.3%)**				
No	82 (90.1%)	16 (72.7%)				
**Hypertension**			5.595	0.22	3.25; 95% CI: 1.18–8.96	**0.018**
Yes	16 (17.6%)	**9 (40.9%)**				
No	75 (82.4%)	13 (59.1%)				

**Legend**: Values are presented as n (%) for categorical variables. *p*-values refer to comparisons between groups using the chi-square (χ^2^) test, the phi coefficient (φ), and the Odds Ratio (OR)﻿

Hypertension was associated with greater COVID-19 severity. A significant association was observed using the Pearson (χ² (1, *N* = 113) = 4.650; *p* = 0.031; φ ≈ 0.20), although Fisher’s exact test did not confirm statistical significance in the two-sided analysis (*p* = 0.072), suggesting a borderline result.

Individuals with diabetes showed higher odds of presenting moderate/severe COVID-19 compared to non-diabetic individuals (OR = 3.42, 95% CI: 1.12–10.46). Although the Pearson chi-square test indicated a significant association (χ² (1) = 4.65, *p* = 0.031), Fisher’s exact test suggested a borderline result (*p* = 0.072), likely due to small, expected cell counts.

No association was observed between obesity and COVID-19 severity (χ² (1, *N* = 112) = 0.025; *p* = 0.874; φ ≈ 0.02). This finding was confirmed by Fisher’s exact test (*p* = 1.000). The odds of moderate/severe COVID-19 were similar between obese and non-obese individuals (OR = 1.10; 95% CI: 0.33–3.67).

### C-reactive protein (CRP) levels

**Fig. 1:**
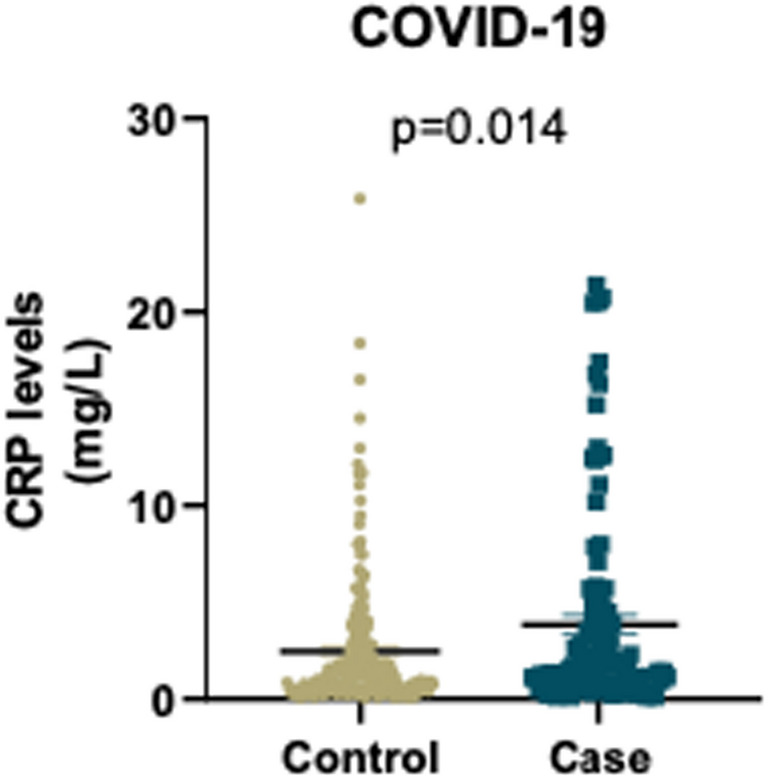
C-reactive protein (CRP) levels in case individuals (post-COVID-19, *n* = 107) and controls (*n* = 226). CRP levels were higher in individuals with post-COVID-19 infection than in controls (*p* = 0.014)

**Fig. 2:**
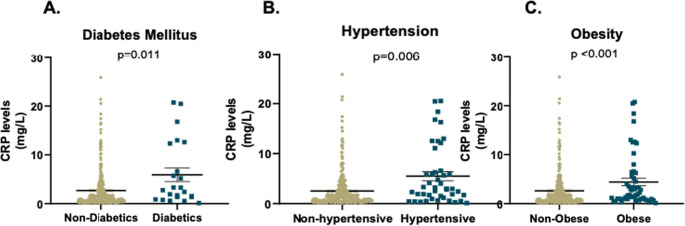
C-reactive protein (CRP) levels in case individuals (post-COVID-19) and controls who had some comorbidity vs. those who had not. non-diabetics (*n* = 310), diabetics (*n* = 22); non-hypertensive (*n* = 279), hypertensive (*n* = 53); non-obese (*n* = 289), obese (*n* = 42). CRP levels were higher in individuals with diabetes (*p* = 0.011, A), hypertension (*p* = 0.006, B), and obesity (*p* < 0.001, C) than in individuals without comorbidities

Serum CRP levels were significantly higher in the Case (post-COVID-19) group compared to the Control group (*p* = 0.014; Fig. 1). Furthermore, within the Case and Control groups, individuals with comorbidities had higher CRP levels than those without comorbidities. This increase was statistically significant for diabetes (*p* = 0.011; Fig. 2A), hypertension (*p* = 0.006; Fig. 2B), and obesity (*p* < 0.001; Fig. 2C). Although these differences were statistically significant, their magnitude should be interpreted in conjunction with the overall clinical context and the multifactorial nature of systemic inflammation.

### Psychiatric Symptoms

**Fig. 3:**
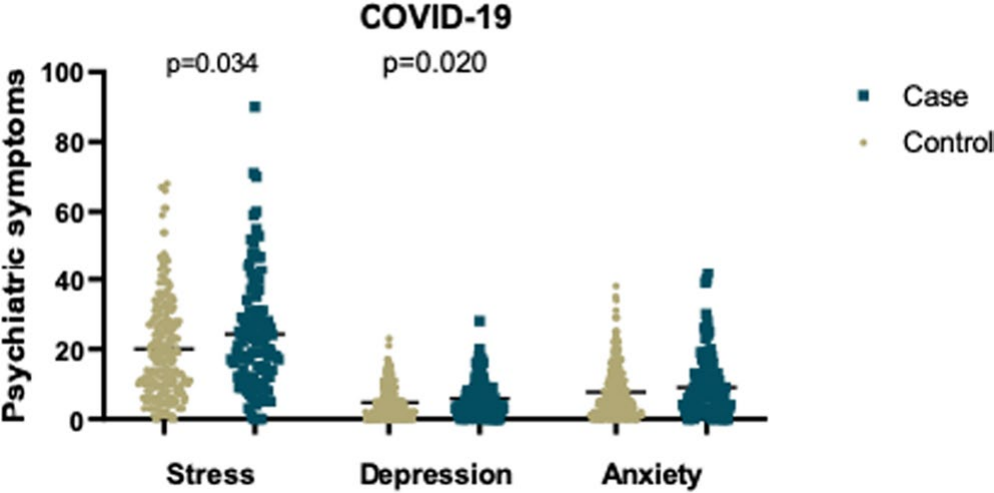
Psychiatric symptoms in individuals post-COVID-19 (cases) and controls. The case (post-COVID-19) group presented significantly higher stress levels (*p* = 0.034) and greater severity of depressive symptoms (*p* = 0.020) than the Control group

Regarding mental health, the Case (post-COVID-19) group presented significantly higher stress levels (*p* = 0.034) and greater severity of depressive symptoms (*p* = 0.020) than the Control group. There was no significant difference in the severity of anxious symptoms between the groups (*p* = 0.109; Fig. 3).

**Fig. 4:**
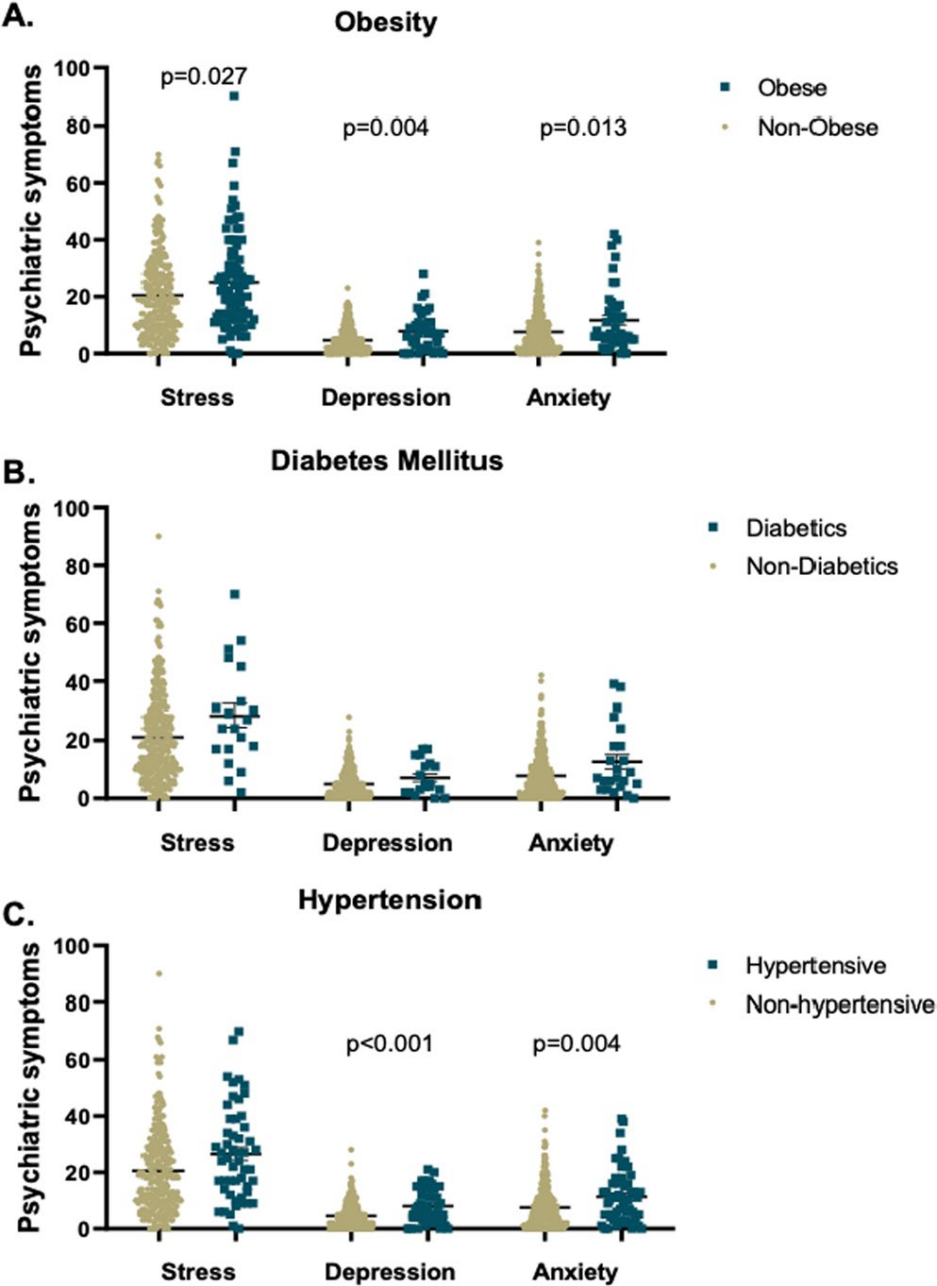
Psychiatric symptoms in case individuals (post-COVID-19) and controls who had some comorbidity vs. those who had not. Obese individuals exhibited greater severity of depressive (*p* = 0.004) and anxious (*p* = 0.013) symptoms, and higher stress levels (*p* = 0.027) than non-obese individuals (**A**). Hypertensive individuals presented greater severity of depressive (*p* < 0.001) and anxious (*p* = 0.004) symptoms, and stress levels (*p* = 0.012) compared to non-hypertensive individuals (**C**)

When stratifying the analysis by the presence of comorbidities in the entire sample, there were no significant differences in the severity of depressive (*p* = 0.112) and anxious (*p* = 0.078) symptoms, or in stress levels (*p* = 0.056), between diabetics and non-diabetics (Fig. 4A). Hypertensive individuals presented significantly greater severity of depressive (*p* < 0.001) and anxious (*p* = 0.004) symptoms, in addition to higher stress levels (*p* = 0.012) compared to non-hypertensive individuals (Fig. 4B). Obese individuals also exhibited greater severity of depressive (*p* = 0.004) and anxious (*p* = 0.013) symptoms, and higher stress levels (*p* = 0.027) than non-obese individuals (Fig. 4C). The observed differences were modest and should be interpreted considering both statistical significance and clinical relevance.

## Correlations

Spearman’s correlation analysis (Table 3) revealed significant positive associations in the post-COVID-19 group. Specifically, hypertension correlated positively with the presence of diabetes, obesity, CRP levels, anxiety symptoms, and stress levels. Obesity correlated positively with CRP levels. Finally, CRP levels themselves showed a significant positive correlation with the severity of depressive and anxious symptoms and stress levels. Statistical results are reported in Table 3.

**Table 3:** Spearman correlations between comorbidities, CRP, and neuropsychiatric symptoms in the Case group

	Hypertension	Diabetes	Obesity	CRP	Depressive Symptoms	Anxious Symptoms	Stress Levels
* Hypertension*	-	*r* = 0,546** *p* < 0.001	*r* = 0.198* *p* = 0.036	*r* = 0.280** *p* = 0.004	*r* = 0.143 *p* = 0.151	*r* = 0.219* *p* = 0.049	*r* = 0.049 *p* = 0.020
*Diabetes*	*r* = 0,546** *p* < 0.001	-	*r* = 0.090 *p* = 0.343	*r* = 0.189 *p* = 0.053	*r* = 0.113 *p* = 0.304	*r* = 0.104 *p* = 0.229	*r* = 0.054 *p* = 0.580
*Obesity*	*r* = 0.198* *p* = 0.036	*r* = 0.090 *p* = 0.343	-	*r* = 0.259** *p* = 0.008	*r* = 0.104 *p* = 0.300	*r* = 0.153 *p* = 0.105	*r* = 0.154 *p* = 0.113
*CRP*	*r* = 0.280** *p* = 0.004	*r* = 0.189 *p* = 0.053	*r* = 0.259** *p* = 0.008	-	*r* = 0.216* *p* = 0.035	*r* = 0.232* *p* = 0.016	*r* = 215* *p* = 0.023
*Depressive Symptoms*	*r* = 0.143 *p* = 0.151	*r* = 0.113 *p* = 0.304	*r* = 0.104 *p* = 0.300	*r* = 0.216* *p* = 0.035	-	*r* = 795** *p* < 0.001	*r* = 0.716** *p* < 0.001
*Anxious Symptoms*	*r* = 0.219* *p* = 0.049	*r* = 0.104 *p* = 0.229	*r* = 0.153 *p* = 0.105	*r* = 0.232* *p* = 0.016	*r* = 795** *p* < 0.001	-	*r* = 0.747** *p* < 0.001
*Stress Levels*	*r* = 0.049 *p* = 0.020	*r* = 0.054 *p* = 0.580	*r* = 0.154 *p* = 0.113	*r* = 215* *p* = 0.023	*r* = 0.716** *p* < 0.001	*r* = 0.747** *p* < 0.001	-

### Multivariate regression analyses

Multivariate linear regression analyses were performed with CRP levels as the dependent variable, adjusting for sex, age, years of schooling, smoking status, COVID-19 medication use, and COVID-19 severity.

Female sex and lower educational level remained independently associated with higher CRP levels across all adjusted models. Anxiety symptoms demonstrated a marginal association with CRP levels after adjustment for confounding variables (β = 0.165, *p* = 0.090). Stress levels also showed a marginal association with CRP levels in the adjusted analyses (β = 0.160, *p* = 0.093). Depressive symptoms were not independently associated with CRP levels after multivariate adjustment (β = 0.142, *p* = 0.147). COVID-19 severity was not independently associated with CRP levels in the adjusted models (*p* > 0.05). Years of education remained inversely associated with CRP levels across all models (β ranging from − 0.282 to -0.328; p ranging from 0.001 to 0.005). Sex remained independently associated with CRP levels across all adjusted models (β ranging from − 0.182 to -0.209; p ranging from 0.033 to 0.046) (Table 4).

**Table 4:** Multivariate linear regression models for CRP levels

Variables	Model 1 β (*p*-value)	Model 2 β (*p*-value)	Model 3 β (*p*-value)	Model 4 β (*p*-value)
Sex	-0.246 (0.009)	-0.197 (0.046)	-0.206 (0.033)	-0.209 (0.037)
Age	0.182 (0.075)	0.136 (0.188)	0.190 (0.062)	0.235 (0.024)
Years of education	-0.289 (0.005)	-0.282 (0.005)	-0.328 (0.001)	-0.294 (0.005)
Smoking status	0.051 (0.580)	0.056 (0.542)	0.037 (0.689)	0.039 (0.680)
COVID-19 medication use	0.090 (0.321)	0.064 (0.482)	0.076 (0.408)	0.006 (0.946)
COVID-19 severity	—	0.113 (0.230)	0.098 (0.301)	—
HAM-A	—	0.165 (0.090)	—	—
Stress levels	—	—	0.160 (0.093)	—
HAM-D	—	—	—	0.142 (0.147)

**Legend**: Values are presented as standardized beta coefficients (β) and p-values. Model 1 included demographic and clinical confounding variables. Model 2 included anxiety symptoms (HAM-A). Model 3 included stress levels. Model 4 included depressive symptoms (HAM-D). All regression models were adjusted for sex, age, years of education, smoking status, COVID-19 medication use, and COVID-19 severity

Overall, the adjusted associations were modest, suggesting that inflammatory status represents one of several factors associated with neuropsychiatric symptoms following COVID-19.

## Discussion

The results of this cross-sectional study demonstrate an interesting association between previous SARS-CoV-2 infection, an elevated inflammatory state (reflected by CRP), the presence of cardiometabolic comorbidities (SAH, diabetes, and obesity), and the exacerbation of neuropsychiatric symptoms, particularly depression, anxiety, and stress. After adjustment for potential confounding variables, the associations between CRP levels and psychiatric symptoms were attenuated. Anxiety symptoms and stress levels remained marginally associated with CRP, whereas depressive symptoms were no longer independently associated. These findings suggest that sociodemographic and clinical factors may partially explain the relationship between inflammation and psychiatric symptoms in individuals with previous COVID-19 infection.

Our adjusted models showed that female sex and lower educational attainment were independently associated with higher plasma CRP levels. The finding that female participants exhibited higher baseline inflammation aligns with known sexual dimorphism, in which females generally mount more robust immune responses. This process, while protective during acute viral clearance, may predispose them to persistent low-grade systemic inflammation (Klein and Flanagan 2016).

Additionally, the inverse relationship between years of education and CRP levels highlights the impact of socioeconomic factors on biology. Lower educational levels are often associated with chronic stressors, limited access to healthcare, and lower health literacy, which can hinder the management of metabolic risk factors (Sen et al. 2023). Therefore, educational attainment may serve as a proxy for social determinants of health that influence baseline immune function, independently of initial COVID-19 clinical severity (Leveau and Velázquez 2024).

Beyond direct pathophysiological pathways, these findings should be interpreted considering the social and behavioral changes caused by the pandemic. Prolonged social isolation, economic instability, and stress acted as significant environmental factors that forced lifestyle modifications (Woods et al. 2023). For many individuals, particularly those in vulnerable socioeconomic situations, these disruptions led to sedentary behavior, poor dietary patterns, and sleep fragmentation, which can independently increase systemic inflammatory markers like CRP (Rivera et al. 2021). In individuals with pre-existing obesity, hypertension, or diabetes, these behavioral shifts likely aggravated metabolic states, linking psychological distress with metabolic inflammation (Callender et al. 2020). Therefore, neuropsychiatric symptoms post-COVID-19 reflect a combination of viral immune triggers, chronic metabolic disease, and pandemic-related psychosocial factors.

In this context, our findings support recent evidence positioning COVID-19 as a systemic condition with significant metabolic, cardiovascular, and mental health repercussions (Rogers et al. 2020; de Azevedo Cardoso et al. 2023). The higher prevalence of obesity, DM, and SAH in the post-COVID-19 group highlights the vulnerability of these patients, both to acute infection and its long-term sequelae. This aligns with literature identifying these comorbidities as independent risk factors for COVID-19 severity and mortality (Santos et al. 2021; Armstrong et al. 2022). Moreover, the chronic low-grade inflammation characteristic of these conditions may exacerbate the immune response to viral infections, perpetuating systemic inflammatory cycles (Hussman 2020).

The significant elevation of CRP in the post-COVID-19 group, especially among those with comorbidities, confirms the persistence of immune activation after the acute phase. CRP was selected in this study due to its stability, clinical reproducibility, and widespread availability as a standardized marker of systemic inflammation (Pepys and Hirschfield 2003). As established, CRP is primarily induced by IL-6 and serves as a key marker of the inflammatory cascade (Toniatti et al. 1990). Although evaluating a broader panel of cytokines could provide a more comprehensive assessment, the positive correlation observed between CRP levels and the severity of depressive, anxious, and stress symptoms supports the association between systemic inflammation and mood disorders. However, although statistically significant, the observed associations were generally modest after adjustment for potential confounding variables, indicating that systemic inflammation should be interpreted as one component of a multifactorial process rather than an isolated determinant of neuropsychiatric symptoms. Mechanistically, peripheral inflammatory mediators can compromise the blood-brain barrier, promoting neuroinflammation through microglial activation (Sproston and Ashworth 2018). This activation can alter neurotransmitter pathways and cause hypothalamic–pituitary–adrenal (HPA) axis dysregulation, which is closely linked to affective symptoms (Dantzer et al. 2008; Howren et al. 2009; Osimo et al. 2019; Mac Giollabhui et al. 2025).

The specific association of SAH and obesity with the worsening of psychiatric symptoms, in contrast to the lack of a significant association for DM in this study, is a relevant finding. Both SAH and obesity share inflammatory and oxidative stress pathophysiological pathways that directly impact endothelial function and cerebral homeostasis (Fernández-Sánchez et al. 2011; Hall et al. 2015).

Regarding the diabetes results, the lack of a statistically significant association with neuropsychiatric symptoms warrants careful interpretation. This outcome may reflect limited statistical power due to the relatively small sample size of diabetic individuals within our stratified cohorts. Alternatively, it could be attributed to unmeasured variability in glycemic control or medication adherence among participants, both of which significantly influence neuroinflammatory pathways (Semenkovich et al. 2015). Additionally, exploring alternative biomarkers beyond CRP could help clarify the specific correlation between metabolic dysregulation and neuropsychiatric outcomes in future research (Ajele and Idemudia 2025).

Specifically, the bidirectional relationship between hypertension and depression is increasingly recognized, involving mechanisms such as autonomic dysfunction, stress axis activation, and systemic inflammation (Satapathy et al. 2025). Similarly, obesity promotes the release of proinflammatory adipokines that can exacerbate central neuroinflammation (Fernández-Sánchez et al. 2011). Furthermore, literature indicates high prevalences of anxiety and depression in COVID-19 survivors, which are often linked to the initial severity of the infection and pre-existing comorbidities (Rocha et al. 2025). The post-pandemic increase in DM and SAH prevalence, potentially aggravated by behavioral changes during isolation, suggests that the pandemic acted as a catalyst for metabolic and cardiovascular decompensation (Chiavarini et al. 2024; Ogungbe et al. 2025).

Additionally, our findings revealed that the severity of acute clinical manifestations of SARS-CoV-2 infection was significantly higher among individuals with DM and SAH, who presented with a significantly higher prevalence of moderate and severe symptoms. This association corroborates the literature identifying T2DM and cardiovascular diseases as independent predictors of poorer prognosis and unfavorable outcomes during the acute phase of COVID-19, including an increased risk of respiratory failure in hypertensive individuals (Hu et al. 2026; Klaudel et al. 2025). Pathophysiologically, the pre-existing endothelial dysfunction and chronic cardiovascular remodeling characteristic of SAH, added to the glycotoxic stress and dysregulated immune response of T2DM, establish a highly susceptible cellular environment that amplifies both the viral-damaging potential and the upstream inflammatory cascade (Manoukian et al. 2026; Pawluszkiewicz et al. 2026).

While this study emphasizes biological inflammation, neuropsychiatric outcomes in post-COVID-19 individuals are closely linked to psychosocial factors. The broader context of the pandemic, including social isolation, bereavement, occupational instability, and chronic stress, played an undeniable role in compounding mental health vulnerabilities. This underscores the necessity of a holistic biopsychosocial framework to avoid overemphasizing biological determinism at the expense of environment-driven psychological distress (Holmes et al. 2020).

Despite these insights, this study has limitations. The cross-sectional design precludes inference about causality or directionality in the observed relationships, meaning that reverse causation and bidirectional relationships among COVID-19 infection, inflammation, comorbidities, and neuropsychiatric symptoms cannot be ruled out. In addition, information on hospitalization, intensive care unit admission, and oxygen requirement was not recorded, which could influence inflammatory markers such as CRP. CRP is a nonspecific inflammatory marker. Self-reported data on comorbidities may introduce bias. Psychiatric history, physical inactivity, socioeconomic adversity, and specific medication classes, which were not evaluated, could also influence the results. Although multivariate analyses were performed, residual confounding cannot be completely excluded. No formal correction for multiple comparisons was applied, which may increase the risk of type I error. However, given the exploratory nature of the study, findings were interpreted cautiously, emphasizing overall patterns rather than isolated significant results. Finally, the convenience sample may limit the generalization of the results. Notwithstanding these limitations, the clinical implications are significant. The findings reinforce the need for a comprehensive and multidisciplinary care approach for COVID-19 survivors, especially those with pre-existing comorbidities. Post-COVID follow-up protocols should systematically incorporate screening for depressive and anxious symptoms, assessment of the inflammatory profile (such as CRP dosage), and aggressive management of metabolic and cardiovascular comorbidities.

## Conclusion

The present findings support an association between previous SARS-CoV-2 infection, systemic inflammation, metabolic and cardiovascular comorbidities, and neuropsychiatric symptoms. However, the observed effect sizes were generally modest, indicating that inflammation represents one of multiple factors potentially involved in these outcomes. Longitudinal studies including additional inflammatory biomarkers are warranted to clarify causal pathways and the clinical relevance of these associations.

## Data Availability

No datasets were generated or analysed during the current study.
